# Diagnostic Blood-Based Biomarkers of Amyloid-β and Tau Pathologies Prior to Alzheimer’s Disease Diagnosis: a Rapid Umbrella Review

**DOI:** 10.1007/s42399-026-02319-6

**Published:** 2026-04-06

**Authors:** Negar Yousefzadeh, Oshin Sharma, Aoife Oliver, Hannah O’Keefe, Emily G. Robertson, Bethan Harris, Gemma Frances Spiers, Dawn Craig

**Affiliations:** NIHR Innovation Observatory, Population Health Sciences Institute, Newcastle University, Newcastle Upon Tyne, UK

**Keywords:** Alzheimer's disease, Biomarkers, Amyloid beta-peptides, Tau proteins

## Abstract

**Background:**

Amyloid-β plaques and tau tangles are established hallmarks of Alzheimer’s disease (AD). Early detection of these pathological changes in preclinical and prodromal stages can enable timely intervention and improve outcomes. This umbrella review synthesises evidence from systematic reviews examining diagnostic blood-based biomarkers (BBMs) predictive of amyloid-β and tau pathologies prior to clinical AD diagnosis.

**Methods:**

We conducted an umbrella review of systematic reviews published between 2018 and 2024, selecting those that synthesised data on BBMs associated with amyloid-β or tau pathologies in adults in preclinical or prodromal AD stages. Searches were performed across Medline, Embase, Cochrane databases, CINAHL, Web of Science, Epistemonikos, and grey literature. A narrative synthesis approach was used. AMSTAR2 was applied for quality appraisal.

**Results:**

Eighteen systematic reviews were included. Eight reviews were rated high or moderate quality using AMSTAR 2. Across the 18 reviews, 556 primary studies were represented, and overlap was low (38 studies; 6.8%). Forty‑four blood-based biomarkers (BBMs) were reported as associated with amyloid-β and/or tau pathology, but only three reviews reported diagnostic or prognostic performance metrics (e.g., sensitivity/specificity, PPV/NPV or AUC). Evidence with the clearest translational signal supported use of panels combining amyloid measures (e.g., plasma Aβ42/Aβ40 ratio) with APOE4 + status and/or phosphorylated tau, and plasma GFAP as an aid to distinguish amyloid-positive from amyloid-negative individuals in symptomatic populations.

**Conclusions:**

BBMs have the potential to widen access to amyloid and tau pathology assessment earlier in the diagnostic pathway. However, limitations in consistently reported accuracy metrics, heterogeneous populations and assays, and the small number of clinically validated tests mean that clear recommendations for routine clinical implementation cannot yet be made. Future evidence syntheses should prioritise (i) standardised reporting of diagnostic accuracy against reference standards (Aβ-PET/CSF), (ii) head‑to‑head comparisons of leading candidates (p‑tau isoforms, Aβ42/Aβ40, GFAP, NfL) and (iii) evaluation in real‑world diagnostic pathways (primary care, memory clinics).

**Supplementary Information:**

The online version contains supplementary material available at 10.1007/s42399-026-02319-6.

## Introduction

Alzheimer’s disease (AD) accounts for 60%–70% of cases of dementia, globally. According to the World Health Organization, more than 55 million people around the globe are living with dementia. The rising trends suggest that there may be nearly 10 million new cases annually, due to population ageing [1]. AD, as a prevalent form of neurodegenerative dementia, has substantial adverse consequences for patients’ quality of life, and that of their families and caregivers. Additionally, AD imposes a substantial socio-economic burden on national, regional, and global healthcare systems [2]. As AD prevalence continues to rise, the associated costs of care, including resource utilization and productivity loss, are anticipated to increase [3].

Neurodegeneration in Alzheimer’s disease is associated with toxic amyloid‑β (Aβ) oligomers and other protein aggregates, with intra‑neuronal neurofibrillary tangles composed of hyperphosphorylated microtubule‑associated tau [4]. The disease course is commonly described as (i) a preclinical stage with biomarker changes in the absence of overt symptoms, (ii) a prodromal stage (often characterised by mild cognitive impairment, MCI) and (iii) symptomatic dementia. In recent years, advances in analytical platforms (e.g., immunoassays, mass‑spectrometry and ultra‑sensitive digital assays) have improved the feasibility of detecting AD‑related proteins and downstream injury or glial markers in peripheral blood [3]. Moreover, the identification of the risk of developing AD in individuals has entered a new phase through the utilization of microRNA (miRNA) as markers of AD. As minimally invasive diagnostic biomarkers, circulating microRNAs in the blood can expedite and enhance early diagnosis, ongoing monitoring, prognosis, and evaluations of therapeutic interventions [5]. These developments may facilitate earlier identification of individuals with underlying Aβ and/or tau pathology and support triage into more definitive confirmatory testing [4]. In this rapid umbrella review, we synthesise systematic review evidence on blood‑based biomarkers that are associated with Aβ and tau pathology before a clinical AD diagnosis, with a particular focus on which candidates are most frequently evaluated versus which have reported performance metrics against reference standards.

## Methods

Initial scoping of evidence about BBM indicated numerous systematic reviews had already synthesised primary study data in this field. Our approach was therefore to undertake a rapid review of systematic reviews (‘rapid umbrella review’). This method makes good use of existing syntheses in published systematic reviews and minimises the risk of duplication [6].

### Inclusion and Exclusion Criteria

The review criteria are summarised in Table 1. The study population consists of preclinical and prodromal adults who are above 18 years of age. Preclinical Alzheimer’s Disease (AD), representing the asymptomatic phase of the disease, is typically defined by the presence of biomarker changes without the manifestation of clinical symptoms. Moreover, prodromal AD is characterized as the initial symptomatic stage when cognitive symptoms are observable, yet the criteria for diagnosing dementia have not yet been met. For accurate investigation and comparison of different BBMs categories and classifying those diagnostic blood-based biomarkers of Amyloid-β and Tau pathologies prior to Alzheimer’s disease diagnosis, we considered both preclinical and prodromal adults. Initially, we limited reviews to those published between 2018 and 2024. After screening, we further narrowed the scope to reviews published in the last year. This decision was due to the large volume of eligible reviews published in this period and to prioritise the most contemporary evidence syntheses.

**Table 1:** Review eligibility criteria

Population	Exposure	Outcome	Study design and source
Pre-clinical or prodromal adults (18 + years).	Any biomarker investigated through blood-based fluids (plasma, serum)	Diagnostic AD biomarkers characterized by amyloid-β and tau pathologies	Review of review studies, published from January 2018 onwards. Reviews must synthesise evidence about the association between a blood-based biomarker and amyloid-β and tau pathologies.

The primary focus of this investigation is on the diagnostic potential of blood-based biomarkers in detecting amyloid and tau pathologies. It’s important to note that this review does not encompass evaluative evidence concerning the effectiveness or implementation feasibility of blood-based biomarkers as a diagnostic tool for Alzheimer’s disease in clinical practice.

Rationale for focusing on blood-based biomarkers: Cerebrospinal fluid (CSF) biomarkers and amyloid/tau PET are established reference standards for detecting AD pathology, but access is constrained by cost, infrastructure, and the invasiveness of lumbar puncture or exposure to radiation. Blood-based biomarkers offer a minimally invasive, lower‑cost and potentially scalable alternative that could support earlier assessment and triage (e.g., in primary care or memory services) before confirmatory CSF or PET testing. We therefore focussed on blood-based biomarkers, while using PET and/or CSF measures as reference standards were reported in the included systematic reviews.

## Search Strategy

An information specialist designed the search strategy in MEDLINE (Ovid) and translated it to other databases. Searches were first run on 5 July 2023 and updated on 25 July 2024 in MEDLINE (Ovid), Embase (Ovid), Cochrane CENTRAL, Cochrane CDSR, CINAHL (EBSCOhost), Web of Science and Epistemonikos. We limited results to 1 January 2018 onwards and applied a validated systematic‑review filter. Grey literature was searched to reduce publication bias by screening relevant organisational repositories and evidence portals (Alzheimer’s Association virtual library, Alzheimer’s Society, Alzheimer’s Research UK, Dementia UK, Alzheimer Scotland, Age UK, Research Institute for the Care of Older People (RICE), and the UK Dementia Research Institute). For each source we used site‑specific search functions (where available) and manual browsing of evidence/report sections using terms relating to Alzheimer’s disease, biomarkers, blood/plasma/serum, amyloid and tau. Records from grey literature were eligible if they met the same review‑of‑reviews criteria and provided sufficient methodological information to confirm systematic methods. The full search strategy is provided in Appendix A.

## Screening

De-duplicated titles and abstracts were uploaded to a Rayyan screening library and screened independently by two reviewers. The full texts of selected records were retrieved and screened independently by one of the four reviewers against the review criteria. We expanded our inclusion beyond the original 2022–2024 scope to include high quality reviews published from 2018 onwards.

## Data Extraction

A standardised extraction form was designed, piloted and refined. Data were extracted independently by four reviewers and cross‑checked for accuracy, with disagreements resolved by discussion. We extracted: (i) review characteristics (author/year, objectives, databases, date range, number and design of included primary studies); (ii) population and disease stage (preclinical, prodromal/MCI, symptomatic); (iii) index biomarkers (e.g., plasma/serum analytes, exosomal markers) and assay platforms; (iv) reference standards (Aβ-PET, CSF biomarkers, MRI/clinical diagnosis); (v) effect measures and diagnostic performance metrics where reported (sensitivity, specificity, AUC, PPV/NPV, LR+/-); and (vi) funding/conflicts of interest and key methodological strengths and limitations noted by review authors.

## Quality Appraisal

The methodological quality of included studies was assessed by three reviewers using the 16-item “A Measurement Tool to Assess Systematic Reviews” (AMSTAR) 2 appraisal tool [7]. The tool comprises the following domains: [1] inclusion of PICO components (P = Patient/Population, I = Intervention, C = Comparison, O = Outcome); [2] protocol before start of the review; [3] study design selection; [4] comprehensive literature search strategy; [5] duplicate study selection; [6] duplicate data extraction; [7] details of excluded studies; [8] description of included studies; [9] risk of bias assessment; [10] funding sources; [11] appropriate statistical methods; [12] assessment of impact of risk of bias; [13] discussion of impact of risk of bias; [14] heterogeneity; [15] investigation of publication bias; [16] report of conflict of interest. Each domain is judged with “Yes”, “Partial Yes” or “No”. Seven of these domains are considered as critical [2, 4, 7, 9, 11, 13, 15]. The overall confidence in the results of the systematic reviews and meta-analyses can be scored as high (no or only one non-critical weakness), moderate (more than one non-critical weakness), low (one critical flaw with or without non-critical weaknesses) and critically low (more than one critical flaw with or without non-critical weaknesses) [7].

### Assessment of Overlap in Evidence Across Reviews

We assessed the proportion of overlap in review evidence: the number of single studies reported in two or more systematic reviews included in our synthesis. This step allows us to (a) avoid overweighting findings from single studies that are reported across multiple reviews; and (b) make a judgement on the value of eliminating older systematic reviews that report the same evidence as more recently published reviews that reflect a more contemporary evidence base.

## Results

**Fig. 1:**
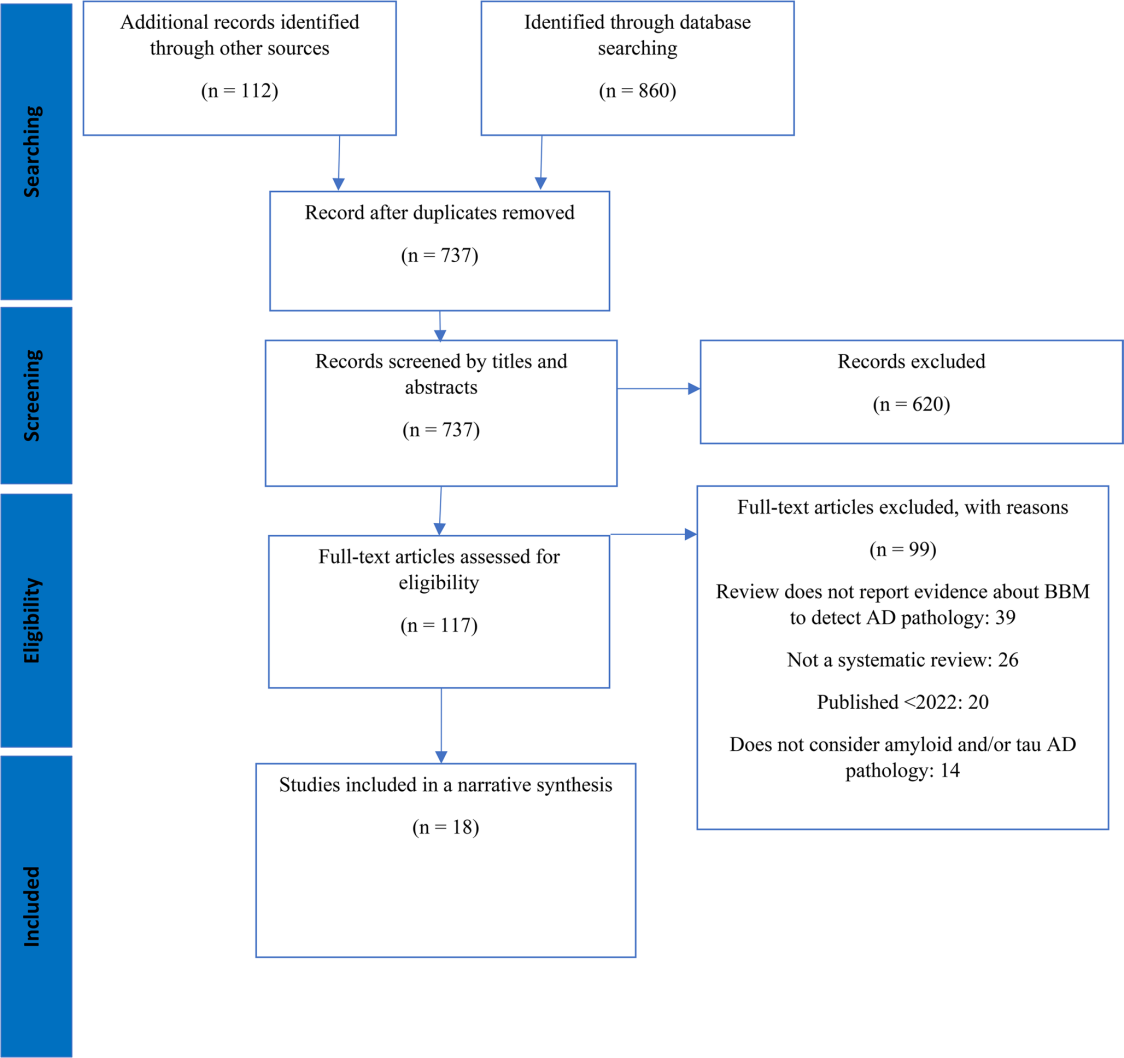
PRISMA Flowchart

We included 18 systematic reviews in the umbrella review (Fig. 1) [8–16]. From each review we extracted: the blood‑based biomarkers evaluated, the AD pathology target (amyloid and/or tau), the reference standard used to define pathology where reported (e.g., Aβ‑PET, CSF biomarkers, clinical conversion), and any reported diagnostic/prognostic accuracy metrics (e.g., sensitivity, specificity, PPV/NPV, AUC).

The characteristics of the included systematic reviews are summarised in Table 2.

**Table 2:** Key features of included studies after the full text screening

Title	Authors	Publication Year	Study design	Stage of Disease	AB BBM	Tau BBM	Other Biomarkers (list)	Age Range	No. of studies in each review
Assessing Adipokines as Potential Biomarkers of Dementia, Alzheimer’s Disease, and Mild Cognitive Impairment: A Systematic Review and Meta-Analysis ^8^	Garcia-Garcia et al.	2023	Systematic Review and Meta-Analysis	Preclinical; Prodromal	Y	Y	four gastrointestinal factors, such as adipokines (e.g., leptin, adiponectin, and resistin) and ghrelin.	47 < x < 83	55 (42 cross-sectional and 13 longitudinal studies)
Association of Circulating Apolipoprotein AI Levels in Patients with Alzheimer’s Disease: A Systematic Review and Meta-Analysis ^9^	Tong et al.	2022	Systematic Review and Meta-Analysis	Preclinical; Prodromal	Y	N	Apolipoprotein AI Levels	60 < X < 85	18
Association of Peripheral Blood Cell Profile with Alzheimer’s Disease ^10^	Huang et al.	2022	Systematic Review and Meta-Analysis	Preclinical; Prodromal	Y	Y	Peripheral blood cell counts and/or lymphocyte subsets	AD: 53 < X < 92.2 HC: 50 < X < 87.4	36
Advances and Applications of Fluids Biomarkers in Diagnosis and Therapeutic Targets of Alzheimer’s Disease ^11^	Xu et al.	2023	Systematic Review	Preclinical; Prodromal	Y	Y	BACE1, NFL, VLIP-1	18 < X	47
Diagnostic Accuracy of Blood-based Biomarker Panels: A Systematic Review ^12^	Hardy-Sosa et al.	2022	Systematic Review	Preclinical; Prodromal	Y	Y	APOEε4	55 < X < 85	76
Emerging Blood Exosome-based Biomarkers for Preclinical and Clinical Alzheimer’s Disease: A Meta-Analysis and Systematic Review ^13^	Liu et al.	2022	Systematic Review and Meta-Analysis	Preclinical, Prodromal and AD	Y	Y	Agouti-related peptide; Angiopoietin 1; BACE-1; BACE1-AS; Cathepsin D; C1q; C4b; C3b; C5b-C9 TCC; CCL5; CD59; CD46; CR1; CSPG4Es; DAF; Factor B-derived fragment Bb; FGF-2; FGF-13; FGF-4; GAP43; HGF; HSP70; IFN-**γ;** IGF-1; IL-1β; IL-2; IL-6; IL-8; γ-secretase; LAMP-1; let-7e-5p; let-7i-5p; LRP6; MBL; miR-15a-5p; miR-15b-3p; miR-18b-5p; miR-20a-5p; miR-23a-3p; miR-30e-5p; miR-93-5p; miR-100-3p; miR-101-3p; miR-106a-5p; miR-106b-5p; miR-125a-5p; miR-125b-5p; miR-126-3p; miR-132; miR-135a; miR-138-5p; miR-139-5p; miR-141-3p; miR-143-3p; miR-150-5p; miR-151a-3p; miR-152-3p; miR-185-5p; miR-190a-5p; miR-193b; miR-204-5p; miR-212; miR-223-3p; miR-23a-3; miR-23b-3p; miR-24-3p; miR-29b-3p; miR-335-5p; miR-338-3p; miR-342-3p; miR-342-5p; miR-361-5p; miR-369-5p; miR-375; miR-384; miR-423-5p; miR-424-5p; miR-548a-5p; miR-582-5p; miR-659-5p; miR-1306-5p; miR-1468-5p; miR-3065-5p; miR-3613-3p; miR-3916; miR-4772-3p; miR-5001-3p; MMP-9; N-(1-carboxymethyl)-L-lysine; Neurogranin; Neuroligin 1; NPTX2; NRXN2; Platelet-derived growth factor BB; P-pan-tyrosine-IRS-1; P-S312-IRS-1; REST; SNAP-25; Synaptotagmin; Synaptopodin; Synaptophysin; TDP-43; Thrombopoietin; Total IRS-1; TNF-α; VEGF-D; VEGFR-2; VEGFR-3	65.95 < X < 74.0	34
GFAP as a Potential Biomarker for Alzheimer’s Disease: A Systematic Review and Meta-Analysis^14^	Kim, K. et al.	2023	Systematic Review and Meta-Analysis	Preclinical; Prodromal	Y	N	GFAP	68.8 < X < 77.78	31
Progression of Subjective Cognitive Decline to MCI or Dementia in Relation to Biomarkers for Alzheimer Disease: A Meta-Analysis^15^	Rostamzadeh et al.	2022	Systematic Review and Meta-Analysis	Preclinical	Y	Y	Aβ42, Aβ42/Aβ−40 ratio, Amyloid PET, Tau PET, P-tau, and Total Tau (t-tau)	60 < X < 78	8
Prospective Biomarkers of Alzheimer’s Disease: A Systematic Review and Meta-Analysis ^16^	Li et al.	2022	Systematic Review and Meta-Analysis	Preclinical and Prodromal	Y	Y	Neurogranin, IL-6, neurofilament light chain	82 < X < 63	84
Serum Glial Fibrillary Acidic Protein is a Body Fluid Biomarker: A Valuable Prognostic for Neurological Disease - A Systematic Review^17^	Heimfarth et al.	2022	Systematic Review	Preclinical	Y	N	Serum glial fibrially acid (GFAP)	29 < X < 68	48
Systematic Review: microRNAs as Potential Biomarkers in Mild Cognitive Impairment Diagnosis ^18^	Ogonowski et al.	2022	Systematic Review	Preclinical and Prodromal	Y	Y	MicroRNA	40 < X < 89	30
Association of soluble TREM2 with Alzheimer’s disease and mild cognitive impairment: a systematic review and meta-analysis^19^	Wang et al.	2024	Systematic Review and Meta-Analysis	Preclinical and Prodromal	Y	Y	Soluble triggering receptor expressed on myeloid cells 2 (TREM2)	55.6 < X < 79.59 (AVG)	36
Plasma Aβ biomarker for early diagnosis and prognosis of Alzheimer’s disease – a systematic review^20^	Ebbese et al.	2023	Systematic Review	AD continuum from cognitively normal controls to dementia	Y	N	Aβ42, Aβ42/Aβ−40 ratio	Adults and older adults	17
Prognostic and Predictive Factors in Early Alzheimer’s Disease: A Systematic Review^21^	João Garcia et al.	2024	Systematic Review	Preclinical	N	Y	APOE4, CSF/plasma p-tau, CSF t-tau, and plasma neurofilament light	Adults and older adults	26
Blood Astrocyte Biomarkers in Alzheimer Disease: A Systematic Review and Meta-Analysis^22^	Hopler et al.	2024	Systematic Review and Meta-Analysis	AD clinical spectrum	Y	N	GFAP and YKL-40	Adults and older adults	36
The performance of plasma phosphorylated tau231 in detecting Alzheimer’s disease: A systematic review with meta-analysis^23^	Xu et al.	2023	Systematic Review and Meta-Analysis	Preclinical and Prodromal (AD, MCI, and CU)	N	Y	P-tau231	Adults and older adults	11
A meta-analysis of neurogenic exosomes in the diagnosis of Alzheimer’s disease^24^	Zhang et al.	2023	Systematic Review and Meta-Analysis	Preclinical and Prodromal (AD or MCI)	Y	Y	Aβ42, T-tau, and P-tau181	Adults and older adults	13
miRNAs in cerebrospinal fluid associated with Alzheimer’s disease: A systematic review and pathway analysis using a data mining and machine learning approach^25^	Pereira et al.	2024	Systematic Review	Preclinical, Prodromal and AD	Y	Y	miRNA-30a-3p, miRNA-193a-5p, miRNA-143-3p, miRNA-145-5p	Adults and older adults	24

### Overlap Between Systematic Reviews

Supplementary file A shows the overlap of primary studies across the included systematic reviews. In total, 556 primary studies were represented across the 18 systematic reviews. Only 38 studies (6.8%) appeared in more than one review, indicating low overlap and a low risk of overweighting single primary studies in the narrative synthesis.

## Size and Scope of Evidence about BBM to Detect AD

A total of 556 unique studies across 18 systematic reviews confirms the vast size of this evidence base published within the last two years alone. Not only is the size of this evidence base notable, but so is the scope in terms of the range of biomarker technologies appraised. The 556 unique studies synthesised in these 18 systematic reviews report evidence about 160 biomarkers. Certain BBMs (such as Aβ42, p-tau, t-tau) are seen as more conventional markers and are appraised in multiple reviews as part of the general amyloid/tau pathology. However, with an emphasis on early AD and MCI, systematic reviews typically considered unique and less common biomarkers.

### Quality of the Evidence

AMSTAR 2 assessments are provided in Appendix B. Of the 18 included reviews, six were rated high quality [8–10, 24–26], and two were rated moderate quality [12, 15]; the remaining 10 reviews were rated low (*n* = 4) or critically low (*n* = 6) confidence [11, 13, 16–23]. To minimise the risk that conclusions are driven by methodologically weaker syntheses, our primary narrative synthesis prioritised the eight high/moderate reviews, with all 18 reviews used to map the breadth of candidate biomarkers. Findings from the lower‑confidence reviews largely echoed the same core candidates (amyloid ratios, p‑tau isoforms, GFAP), but typically 

### Which BBM are Promising to Detect AD Pathology?

Across the eight high/moderate‑quality reviews, forty‑four BBMs were reported as associated with amyloid and/or tau pathology (Table 3).

**Table 3:**
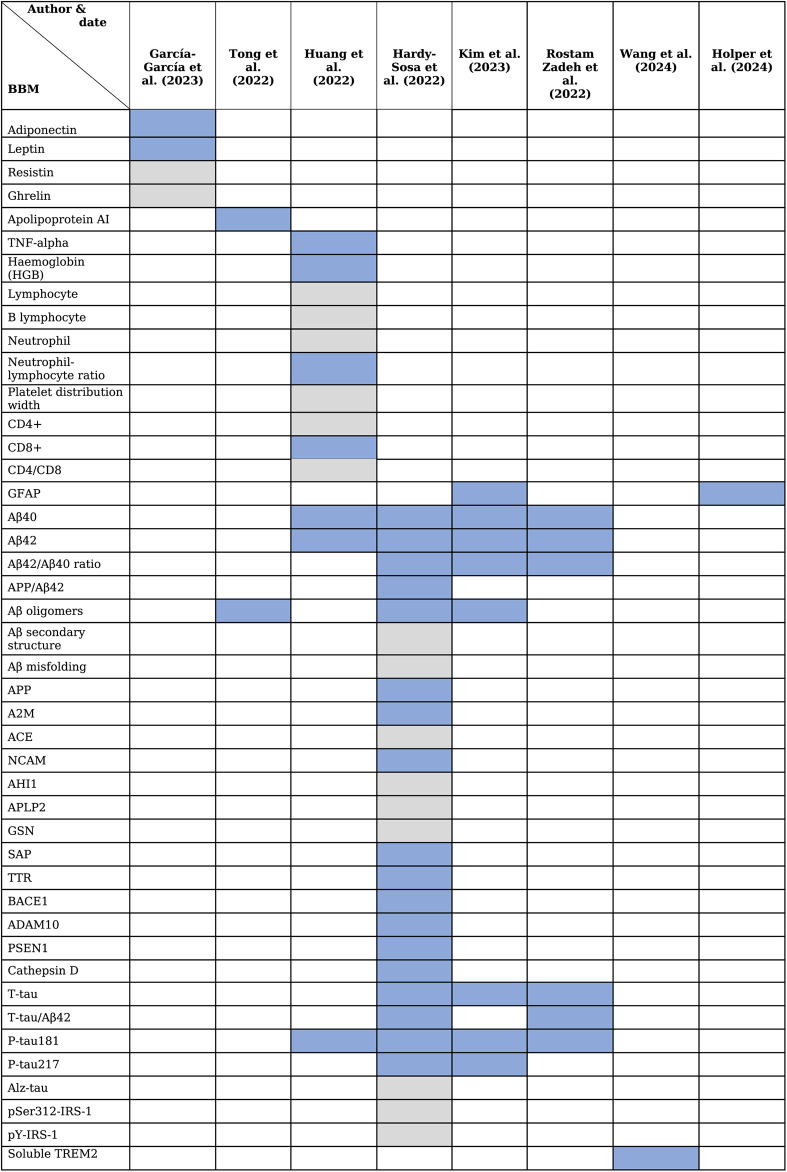
Predictability of Biomarkers of the AB and Tau pathologies in the reviews

Table 4 summarises the BBMs considered predictive, grouped by the pathology target with which they were associated (amyloid‑β, tau, or both).

**Table 4:** Diagnostic blood-based biomarkers of Amyloid-β and Tau pathologies prior to AD

Author and date	Category	Diagnostic BBMs
Hardy-Sosa, et al. (2022)	AB and Tau pathologies	- Aβ40 - Aβ42 - Aβ42/Aβ40 - APP/Aβ42 - AB oligomers - AB secondary structure - AB misfolding - APP - A2M - ACE - NCAM - AHI1 - APLP2 - GSN - SAP - TTR - APP metabolism: BACE1, ADAM10, PSEN1, cathepsin D - t-tau - t-tau/Aβ42 - p-tau181 - Alz-tau R - pSer312-IRS-1 - pY-IRS-1
Tong, et al. (2022)	AB pathology	- Apolipoprotein AI (ApoA-I) - AB oligomers
Huang, et al. (2022)	AB and Tau pathologies	- TNF-a - NLR - Haemoglobin level - CD8 + T - AB oligomers - Total tau (t-tau) - (P)-tau
García-García, et al. (2023)	AB and Tau pathologies	- Leptin - Adiponectin
Kim, et al. (2023)	AB and Tau pathologies	- Glial fibrillary acidic protein (GFAP) - Amyloid beta-42 (Aβ42) - Amyloid beta-40 (Aβ40) - AB oligomers - Total tau (t-tau) - p-tau181 - p-tau217 - the Aβ42/Aβ40 ratio
Rostamzadeh, et al. (2022)	AB and Tau pathologies	- Amyloid beta-42 (Aβ42) - Amyloid beta-40 (Aβ40) - Total tau (t-tau) - p-tau181 - the Aβ42/Aβ40 ratio
Wang, et al. (2024)	AB and Tau pathologies	- Soluble TREM2 (sTREM2)
Holper, et al. (2024)	AB and Tau pathologies	- Glial fibrillary acidic protein (GFAP)

Neutrophil-to-lymphocyte ratio.

Amyloid Precursor Protien(APP).

#### Promising Versus Frequently Evaluated Biomarkers

Seven reviews explicitly recommended using multi‑analyte panels rather than a single biomarker for earlier‑stage assessment (preclinical/prodromal) [8, 11, 12, 16, 18, 24, 26]. Across the evidence base, the most frequently evaluated biomarkers were amyloid measures (Aβ42, Aβ40 and Aβ42/Aβ40 ratio; 7 reviews), tau measures (t‑tau and p‑tau181; 6 reviews) and, less consistently, glial activation markers such as GFAP [8–10, 12, 15, 24, 26]. Frequency of evaluation does not equate to clinical validation.

We therefore grouped biomarkers into evidence tiers:

Tier 1 (performance data reported): three reviews reported diagnostic or prognostic performance metrics. Hardy‑Sosa et al. reported that plasma Aβ42/Aβ40 ratio combined with APOE4 + status achieved the highest accuracy for predicting Aβ‑PET status [12]. Kim et al. synthesised evidence for plasma GFAP and reported discrimination between Aβ‑positive and Aβ‑negative individuals (with CSF or PET comparators) in symptomatic populations [14]. Rostamzadeh et al. reported prognostic performance for combined amyloid and tau markers in predicting conversion from subjective cognitive decline to MCI or dementia [15].

Tier 2 (replicated across reviews but limited performance reporting): Aβ42/Aβ40 ratio, p‑tau181, p‑tau217 and t‑tau were assessed across multiple systematic reviews and are repeatedly highlighted as biologically plausible candidates, but most reviews did not report sensitivity/specificity or AUC in a consistent way that enables direct clinical translation.

Tier 3 (single‑review or emerging candidates): many additional candidates (e.g., exosome‑derived proteins, microRNAs, immune/metabolic markers) were each reported within one review, indicating emerging research activity but insufficient replication for prioritisation without further validation [18–23].

#### Which BBM have the best Predictive Accuracy?

Predictive accuracy was reported in three systematic reviews. In relation to predicting Aβ-PET status, Hardy-Sosa et al. (2022) reported that the ratio Aβ42/Aβ40 combined with APOE4 + status in plasma demonstrated the highest accuracy [12]. For individuals with AD or MCI, the potential of plasma GFAP in distinguishing the AB positive from AB negative individuals appears to be more accurate than CSF GFAP [14]. Amyloid markers in combination with either p-tau or t-tau correctly predict: the presence of clinical progression in about 59.7% of cases; and the absence of clinical progression in about 89.4% of cases [15].

## Discussion

Considering the substantial burden of Alzheimer’s disease, there is a clear need for accessible tools that can support earlier identification of individuals with underlying amyloid and tau pathology, particularly as disease‑modifying therapies increasingly require evidence of pathology for treatment eligibility [27, 28]. Our findings support a pragmatic view of blood‑based biomarkers as pathway tools rather than stand‑alone diagnostic tests.

### Clinical Utility and use Cases

The most defensible near‑term use case is triage: a blood test could be used in primary care or memory services to “rule out” amyloid pathology (reducing unnecessary referrals and PET/CSF testing) and to prioritise higher‑probability patients for confirmatory testing and specialist review. A second use case is specialist diagnostic support, where plasma biomarkers could complement clinical assessment and help determine whether PET or CSF testing is warranted [8–10, 12, 15, 24, 26]. Clinical utility depends on performance metrics in the intended setting and population; in this umbrella review, only three systematic reviews reported diagnostic/prognostic accuracy in a way that directly informs these decisions, which limits immediate translation [12, 14, 15].

### Evolving Standards of Care

Since our searches were completed, regulatory clearance of blood‑based assays has begun to emerge. In May 2025, the U.S. FDA cleared the Lumipulse G pTau217/β‑Amyloid 1–42 plasma ratio as an aid in diagnosing Alzheimer’s disease in symptomatic adults, and in October 2025 the FDA cleared Roche’s Elecsys pTau181 assay as an aid in the initial assessment in primary care [29, 30]. These milestones underline the importance of moving beyond cataloguing candidate biomarkers toward comparative validation (including head‑to‑head testing), reporting of setting‑specific accuracy (e.g., primary care versus memory clinic), and evaluation of implementation barriers such as laboratory infrastructure and cost‑effectiveness [31].

Overall, candidates with the most consistent biological plausibility and replication across reviews (Aβ42/Aβ40 ratio, p‑tau isoforms and GFAP) are best positioned for pathway evaluation, while the many emerging candidates (e.g., microRNAs, exosome‑derived markers, immune/metabolic panels) require replication and standardised reporting before prioritisation.

## Conclusion

BBMs show promise in detecting amyloid-β and tau pathologies prior to AD diagnosis. However, further validation of BBM panels and consistent reporting of diagnostic metrics are essential to support clinical adoption. Future evidence syntheses should prioritise (i) standardised reporting of diagnostic accuracy against reference standards (Aβ-PET/CSF), (ii) head‑to‑head comparisons of leading candidates (p‑tau isoforms, Aβ42/Aβ40, GFAP, NfL) and (iii) evaluation in real‑world diagnostic pathways (primary care, memory clinics).

### Strengths and Limitations

This review features a comprehensive search and robust quality appraisal. Expanding the inclusion timeframe improved completeness. Across the six high/moderate‑quality reviews, all but one synthesised evidence from both preclinical and prodromal (or symptomatic) populations; only one review limited its synthesis to preclinical populations. Study populations were predominantly older adults (> 50 years), with limited evidence in younger or more diverse cohorts. Although heterogeneity in age, sex/gender and disease stage was frequently noted by review authors, stratified diagnostic accuracy analyses were rarely reported and were not reported consistently enough to summarise quantitatively across reviews. This limits the ability to make population‑specific recommendations (e.g., primary care triage versus specialist diagnosis) and reinforces the need for future primary studies and systematic reviews to report accuracy by disease stage, age bands and sex/gender, using standard reference standards and assay methods.

## Supplementary Information

Below is the link to the electronic supplementary material.

Supplementary Material 1(DOCX 16.0 KB)

Supplementary Material 2(DOCX 23.2 KB)

Supplementary Material 3(DOCX 19.8 KB)

Supplementary Material 4(XLSX 45.5 KB)

## Data Availability

Not applicable. All data extraction forms are included in the appendices. Further details can be provided upon reasonable request.
