# Supplementary material for: Diagnostic Blood-Based Biomarkers of Amyloid-β and Tau Pathologies Prior to Alzheimer’s Disease Diagnosis: a Rapid Umbrella Review
Source: SN Compr Clin Med. 2026 Apr 6;8(1):109. doi: 10.1007/s42399-026-02319-6 (PMC13053545; doi:10.1007/s42399-026-02319-6)
Supplement: Supplementary file 1 — Supplementary Material 1(DOCX 16.0 KB) [file 42399_2026_2319_MOESM1_ESM.docx]

# Appendix A: Search Strategy developed in Medline (Ovid)

| 1. | (pre?clinical adj5 (alzheimer?s or "AD")).ti,ab,kf,kw. |
| --- | --- |
| 2. | (before diagnos* adj5 (alzheimer?s or "AD")).ti,ab,kf,kw. |
| 3. | (pre?diagnos* adj5 (alzheimer?s or "AD")).ti,ab,kf,kw. |
| 4. | ((diagnos* or progess* or prospect* or suspect* or prognos* or declin* or degenerat* or impair*) adj5 (alzheimer?s or "AD" or dementia or congnit*)).ti,ab,kf,kw. |
| 5. | or/1-4 |
| 6. | ((blood or serum or plasma or panel or test* or diagnos* or prospective or research or screen*) adj5 biomarker*).ti,ab,kf,kw. |
| 7. | biomarkers/ or biomarkers, pharmacological/ |
| 8. | or/6-7 |
| 9. | (systematic review or meta-analysis).pt. |
| 10. | meta-analysis/ or systematic review/ or systematic reviews as topic/ or meta-analysis as topic/ or "meta analysis (topic)"/ or "systematic review (topic)"/ or exp technology assessment, biomedical/ or network meta-analysis/ |
| 11. | ((systematic* adj3 (review* or overview*)) or (methodologic* adj3 (review* or overview*))).ti,ab,kf. |
| 12. | ((quantitative adj3 (review* or overview* or synthes*)) or (research adj3 (integrati* or overview*))).ti,ab,kf. |
| 13. | ((integrative adj3 (review* or overview*)) or (collaborative adj3 (review* or overview*)) or (pool* adj3 analy*)).ti,ab,kf. |
| 14. | (data synthes* or data extraction* or data abstraction*).ti,ab,kf. |
| 15. | (handsearch* or hand search*).ti,ab,kf. |
| 16. | (mantel haenszel or peto or der simonian or dersimonian or fixed effect* or latin square*).ti,ab,kf. |
| 17. | (met analy* or metanaly* or technology assessment* or HTA or HTAs or technology overview* or technology appraisal*).ti,ab,kf. |
| 18. | (meta regression* or metaregression*).ti,ab,kf. |
| 19. | (meta-analy* or metaanaly* or systematic review* or biomedical technology assessment* or bio-medical technology assessment*).mp,hw. |
| 20. | (medline or cochrane or pubmed or medlars or embase or cinahl).ti,ab,hw. |
| 21. | (cochrane or (health adj2 technology assessment) or evidence report).jw. |
| 22. | (comparative adj3 (efficacy or effectiveness)).ti,ab,kf. |
| 23. | (outcomes research or relative effectiveness).ti,ab,kf. |
| 24. | ((indirect or indirect treatment or mixed-treatment or bayesian) adj3 comparison*).ti,ab,kf. |
| 25. | (multi* adj3 treatment adj3 comparison*).ti,ab,kf. |
| 26. | (mixed adj3 treatment adj3 (meta-analy* or metaanaly*)).ti,ab,kf. |
| 27. | umbrella review*.ti,ab,kf. |
| 28. | (multi* adj2 paramet* adj2 evidence adj2 synthesis).ti,ab,kf. |
| 29. | (multiparamet* adj2 evidence adj2 synthesis).ti,ab,kf. |
| 30. | (multi-paramet* adj2 evidence adj2 synthesis).ti,ab,kf. |
| 31. | or/9-30 |
| 32. | 5 and 8 and 31 |
| 33. | limit 32 to yr="2018-Current" |
