# Supplementary material for: Diagnostic Blood-Based Biomarkers of Amyloid-β and Tau Pathologies Prior to Alzheimer’s Disease Diagnosis: a Rapid Umbrella Review
Source: SN Compr Clin Med. 2026 Apr 6;8(1):109. doi: 10.1007/s42399-026-02319-6 (PMC13053545; doi:10.1007/s42399-026-02319-6)
Supplement: Supplementary file 2 — Supplementary Material 2(DOCX 23.2 KB) [file 42399_2026_2319_MOESM2_ESM.docx]

**Appendix B: AMSTAR 2 assessment**

| **Criteria**  **Titles** | **AMSTAR 1** | **AMSTAR 2** | **AMSTAR 3** | **AMSTAR 4** | **AMSTAR 5** | **AMSTAR 6** | **AMSTAR 7** | **AMSTAR 8** | **AMSTAR 9** | **AMSTAR 10** | **AMSTAR**  **11** | **AMSTAR 12** | **AMSTAR 13** | **AMSTAR 14** | **AMSTAR 15** | **AMSTAR 16** | **Overall confidence** |
| --- | --- | --- | --- | --- | --- | --- | --- | --- | --- | --- | --- | --- | --- | --- | --- | --- | --- |
| **Advances and applications of fluids biomarkers in diagnosis and therapeutic targets of Alzheimer's disease** | Partial Yes | Partial Yes | YES | Partial Yes | No | YES | No | Partial Yes | No | No | NA | NA | NA | NA | NA | YES | Critically Low |
| **Assessing Adipokines as Potential Biomarkers of Dementia, Alzheimer's Disease, and Mild Cognitive Impairment: A Systematic Review and Meta-Analysis** | YES | YES | YES | YES | YES | YES | YES | Partial Yes | YES | No | YES | YES | YES | YES | YES | YES | High |
| **Association of Circulating Apolipoprotein AI Levels in Patients with Alzheimer's Disease: A Systematic Review and Meta-Analysis** | YES | Partial Yes | YES | YES | YES | YES | YES | YES | YES | No | YES | YES | YES | YES | YES | YES | High |
| **Association of Peripheral Blood Cell Profile with Alzheimer's Disease: A Meta-Analysis** | YES | Partial Yes | YES | YES | YES | YES | YES | YES | YES | No | YES | YES | YES | YES | YES | YES | High |
| **Diagnostic Accuracy of Blood-based Biomarker Panels: A Systematic Review** | Partial Yes | Partial Yes | YES | Partial Yes | YES | YES | YES | No | Yes | No | NA | NA | NA | NA | NA | YES | Moderate |
| **Emerging Blood Exosome-based Biomarkers for Preclinical and Clinical Alzheimer's Disease: A Meta-Analysis and Systematic Review** | Partial Yes | YES | No | Partial Yes | YES | YES | YES | Partial Yes | Partial Yes | No | YES | YES | YES | YES | No | YES | Low |
| **GFAP as a Potential Biomarker for Alzheimer's Disease: A Systematic Review and Meta-Analysis** | Partial Yes | Partial Yes | Yes | Partial Yes | YES | YES | YES | YES | YES | No | Partial Yes | YES | YES | YES | YES | YES | High |
| **Progression of Subjective Cognitive Decline to MCI or Dementia in Relation to Biomarkers for Alzheimer Disease: A Meta-Analysis** | YES | YES | No | YES | YES | YES | YES | Partial Yes | Partial Yes | No | YES | YES | Yes | YES | YES | YES | Moderate |
| **Prospective Biomarkers of Alzheimer's Disease: A Systematic Review and Meta-Analysis** | Partial Yes | YES | No | YES | YES | YES | YES | Partial Yes | Partial Yes | No | Partial Yes | YES | No | YES | YES | YES | Low |
| **Serum Glial Fibrillary Acidic Protein is a Body Fluid Biomarker: A Valuable Prognostic for Neurological Disease - A Systematic Review** | Partial Yes | No | No | YES | YES | YES | YES | Partial Yes | No | No | No | No | No | YES | No | YES | Critically low |
| **Systematic Review: microRNAs as Potential Biomarkers in Mild Cognitive Impairment Diagnosis** | Partial Yes | No | No | Partial Yes | No | No | YES | YES | No | No | NA | NA | NA | NA | NA | YES | Critically low |
| **Blood Astrocyte Biomarkers in Alzheimer Disease: A Systematic Review and Meta-Analysis** | YES | YES | YES | YES | YES | YES | YES | YES | YES | YES | YES | YES | YES | YES | YES | No | High |
| **Prognostic and Predictive Factors in Early Alzheimer's Disease: A Systematic Review** | YES | YES | YES | YES | YES | YES | YES | YES | No | No | YES | YES | YES | YES | YES | YES | Low |
| **Plasma AB biomarker for early diagnosis and prognosis of Alzheimer's disease - a systematic review** | Partial Yes | Partial Yes | No | No | YES | YES | YES | YES | No | No | YES | YES | No | YES | YES | YES | Critically low |

- ***Note***: AMSTAR 1: Did the research questions and inclusion criteria for the review include the components of PICO? AMSTAR 2: Did the report of the review contain an explicit statement that the review methods were established prior to the conduct of the review and did the report justify any significant deviations from the protocol? AMSTAR 3: Did the review authors explain their selection of the study designs for inclusion in the review? AMSTAR 4: Did the review authors use a comprehensive literature search strategy? AMSTAR 5: Did the review authors perform study selection in duplicate? AMSTAR 6: Did the review authors perform data extraction in duplicate? AMSTAR 7: Did the review authors provide a list of excluded studies and justify the exclusions? AMSTAR 8: Did the review authors describe the included studies in adequate detail? AMSTAR 9: Did the review authors use a satisfactory technique for assessing the risk of bias in individual studies that were included in the review? AMSTAR 10: Did the review authors report on the sources of funding for the studies included in the review? AMSTAR 11: If meta-analysis was performed, did the review authors use appropriate methods for statistical combination of results? AMSTAR 12: If meta-analysis was performed, did the review authors assess the potential impact of risk of bias in individual studies on the results of the meta-analysis or other evidence synthesis? AMSTAR 13: Did the review authors account for risk of bias in individual studies when interpreting/discussing the results of the review? AMSTAR 14: Did the review authors provide a satisfactory explanation for, and discussion of, any heterogeneity observed in the results of the review? AMSTAR 15: If they performed quantitative synthesis, did the review authors carry out an adequate investigation of publication bias (small study bias) and discuss its likely impact on the results of the review? AMSTAR 16: Did the review authors report any potential sources of conflict of interest, including any funding they received for conducting the review? NA = not applicable. Red indicates “No”, yellow indicates “Partial Yes” and green indicates “Yes”.
