# Supplementary material for: Diagnostic Blood-Based Biomarkers of Amyloid-β and Tau Pathologies Prior to Alzheimer’s Disease Diagnosis: a Rapid Umbrella Review
Source: SN Compr Clin Med. 2026 Apr 6;8(1):109. doi: 10.1007/s42399-026-02319-6 (PMC13053545; doi:10.1007/s42399-026-02319-6)
Supplement: Supplementary file 3 — Supplementary Material 3(DOCX 19.8 KB) [file 42399_2026_2319_MOESM3_ESM.docx]

**Descriptive summary of included reviews and the search strategies**

Even though we narrowed our analysis to encompass only 6 studies of high and moderate quality, determined by their AMSTAR 2 ratings, the below table provides a comprehensive summary of all 11 included reviews, alongside their respective search strategies.

- Brief descriptive summary of included reviews and the search strategies.

| Row | Xu et al. | Garcia-Garcia et al. | Tong et al. | Huang et al. | Hardy-Sosa et al. | Liu et al. | Kim, K. et al. | Rostamzadeh et al. | Li et al. | Heimfarth et al. | Ogonowski et al. |
| --- | --- | --- | --- | --- | --- | --- | --- | --- | --- | --- | --- |
| Summary | Aimed to study the amyloid‐β (Aβ) plaques, Tau protein abnormal phosphorylation, axon damage, synaptic dysfunction, inflammation, and related hypotheses associated with AD mechanisms | targeting the associations between leptin, adiponectin, resistin, and ghrelin and the prevalence, diagnosis, and prognosis of general dementia, AD, and mild cognitive impairment (MCI) | possibility of ApoA-I as a potential biomarker of AD with levels of ApoA-I in serum, plasma, and cerebrospinal fluid between AD patients and healthy controls (HCs) | associations between peripheral blood cell profiles and AD related dementia at preclinical and prodromal stages | conventional (amyloid-beta and tau) and neuroinflammatory biomarkers, such as amyloid beta-42, amyloid beta-40, total tau, phosphorylated tau-181, and other tau isoforms, were the most represented. | core biomarkers including Aβ1–42, P-T181-tau, P-S396-tau, and T-tau were increased in blood neuron-derived exosomes of preclinical Alzheimer’s disease, mild cognitive impairment, and Alzheimer’s disease patients. | evaluate GFAP in peripheral blood as a biomarker for AD; GFAP level in the blood was higher in the Aβ-positive group than in the negative groups, and in individuals with AD or mild cognitive impairment (MCI) compared to the healthy control which may facilitate the diagnosis and prognosis prior to AD | the potential role of these biomarkers:  - Amyloid beta42 (AB42)  -Amyloid beta40 (AB40)  -Total tau (T-tau)  - (P)-tau181  and  -the AB42/AB40 ratio  before initial  symptom onset and subsequent dementia | Investigating the role of CSF and plasma Aβ42, t-tau, p-tau, tau/Aβ42; peripheral blood t-tau, Aβ42/Aβ40, NFL; whole, left and right HV, EC volume, MTA, 18 F-FDG PET, 11 C-PIB PET; APOE ε4 as a promising prospective biomarker for AD progression | Investigating the association between GFAP levels and the disease being studied, suggesting that elevated GFAP levels are a potentially valuable diagnostic biomarker in the evaluation of different neurological diseases at any stages, particularly at preclinical and prodromal stages | Studied the predictability of miRNAs in serum, plasma, and other body fluids as a promising source of biomarkers before the onset of the AD-related cognitive impairments |
| Search Strategy | focused on amyloid-β  (Aβ) plaques, Tau protein  abnormal phosphorylation, axon damage, synaptic dysfunction, inflammation | adipokines/ghrelin and their origin (i.e., serum, plasma, or CSF) results which were group differences in adipokine levels between dementia, AD, or MCI, and cognitively normal participants | “Apolipoprotein A-I,” “ApoA-I,” “Alzheimer's disease any stage,” “AD,” and “Dementia” | Searched key words such as “Alzheimer,” “Preclinical,” “Prodromal,” “mild cognitive impairment,” “blood cell count,” “lymphocyte subsets,” “neutrophil,” “eosinophil,” “basophil” “lymphocyte,” “NLR,” “monocyte,” “white blood cell,” “leucocyte,” “red blood cell,” “erythrocyte,” “haemoglobin,” “platelet,” “PLR,” “mean corpuscular volume,” “red cell distribution width,” “mean platelet volume,” “platelet distribution width,” “CD4+,” “CD8+,” “CD4+/CD8+,” “CD3+,” “B lymphocyte,” and “NK cell.” | “Alzheimer’s disease,” “biomarker,” “panel” (“combined biomarkers” or “signature” or “model”), “blood,” “accuracy” (“AUC” or “ROC” or “specificity” and “sensibility”), “diagnosis,” “amyloid,” “tau,” “neurodegeneration,” and “neuroinflammation.” | AD, MCI, and preclinical AD - and healthy control subjects; exosomes were from peripheral blood, serum, or plasma, and the possibility of origination from different cell types; were also searched and analysed | (AD [MeSH] or Alzheimer* or “mild cognitive impairment”) and (glial fibrillary acidic protein [MeSH] or GFAP) and (blood, plasma, or serum) Alzheimer* or “mild cognitive impairment” and “glial fibrillary acidic protein” or GFAP, and blood, plasma, or serum. The inclusion criterion was a clinical study using human blood samples to assess the GFAP levels between AD and normal controls, or Aβ-positive and Aβ-negative groups. | One section referred to SCD (subjective cognitive decline, subjective memory disorder, subjective memory impairment, subjective cognitive impairment, subjective memory loss, subjective complaints, memory complaints, cognitive complaints, subjective memory decline, subtle cognitive decline, and preclinical and prodromal stage), the second referred to the AD biomarker status, the third to AD, and the fourth to the possible clinical outcome. | (1) the diagnostic criteria of AD at different preclinical and prodromal stages and mild cognitive impairment (MCI) (2) prospective cohort studies with a progressive event of conversion to MCI or AD, which included the conversion from CN/MCI/SCD/SMI to AD and conversion from CN to MCI; (3) odd ratio (OR)/risk ratio (RR)/ with 95% confidence interval (CI) or equivalent value were available | GFAP¨ OR ¨glial fibrillary acidic protein¨ AND ¨neurological¨ OR ¨neurodegenerative¨ AND ¨plasma¨ OR ¨serum¨ | (“blood,” “serum,” “plasma,” and “exosome”). The following step was considered in the title, abstract, or author keywords; at least one of the keywords mentioned above, in addition, mentions “miRNA” or “preclinical”, “prodromal”, “AD pathologies”, “microRNA”, and “cognitive impairment.” |
